# Empowerment among breast cancer survivors using an online peer support community

**DOI:** 10.1007/s00520-024-09119-5

**Published:** 2024-12-28

**Authors:** Marina Ruiz-Romeo, Laura Ciria-Suarez, Joan C. Medina, Maria Serra-Blasco, Arnau Souto-Sampera, Aida Flix-Valle, Alejandra Arizu-Onassis, Carla Morales Moncada, Cristina Villanueva-Bueno, Vicente Escudero-Vilaplana, Eva Juan-Linares, Cristian Ochoa-Arnedo

**Affiliations:** 1 The Bellvitge Biomedical Research Institute IDIBELL, Psychooncology and Digital Health Group, Hospitalet de Llobregat, Spain; 2 Department of Clinical Psychology and Psychobiology, Universitat de Barcelona, Barcelona, Spain; 3 ICOnnecta’t Digital Health Program, Catalan Institute of Oncology, Hospitalet de Llobregat, Spain; 4 Department of Psychology and Education Sciences, Universitat Oberta de Catalunya, Barcelona, Spain; 5 Pharmacy Service, Gregorio Marañón General University Hospital and Gregorio Marañón Health Research Institute, Madrid, Spain; 6 Psychooncology Unit, Hospital of the Holy Cross and Saint Pau, Barcelona, Spain

**Keywords:** Online communities, Empowerment, Peer support, Breast cancer, Oncology

## Abstract

**Objectives:**

Breast cancer (BC) impacts the patients’ quality of life. Peer support can provide emotional understanding and enhances access to information, social support, coping strategies, and empowerment. *Comunitats* is an online peer support community app for BC survivors that involves healthcare professionals. This study aims to explore how participation in *Comunitats* promotes empowerment, and to identify the variables related to it.

**Methods:**

A prospective, cross-sectional approach was applied. One hundred twenty-one women diagnosed with BC were included in *Comunitats.* Sociodemographic and clinical variables, along with measures of emotional distress (HADS), post-traumatic growth (PTGI), and empowerment (Van Uden-Kraan’s Empowerment Questionnaire), were collected through an online questionnaire completed by the participants. Additionally, data on participation in the online community were obtained directly from the app. Assessments were conducted at inclusion and again 3 months later. Correlations were used to guide linear regression analysis to identify the variables predicting greater empowerment outcomes.

**Results:**

Empowerment assessment indicated that participants felt empowered by their involvement in *Comunitats*. The most commonly experienced empowerment outcomes were “being better informed” and “improved acceptance of the illness.” “Exchanging information” and “finding recognition” were the most strongly experienced empowerment processes and the strongest predictors of empowerment outcomes in the regression analysis.

**Conclusion:**

Involvement in *Comunitats* enhances empowerment in BC survivors. Empowering processes within the community partially predict overall empowerment outcomes.

**Practical implications:**

Empowerment positively impacts self-care autonomy, self-efficacy, and treatment adherence, promoting healthier lifestyles and enhanced treatment outcomes. Therefore, we recommend encouraging participation in online peer support communities, as it might enhance empowerment.

**Supplementary Information:**

The online version contains supplementary material available at 10.1007/s00520-024-09119-5.

## Introduction

Breast cancer (BC) is the most prevalent oncological disease worldwide, with an estimated incidence of 2.3 million diagnoses in 2020, representing 11.7% of cancer diagnoses [1].

The diagnosis of BC, the oncological treatment, and the subsequent adaptation to changes experienced during the process can have a negative impact on quality of life (QoL), including physical, emotional, social, and functional areas [2, 3]. Nevertheless, several psycho-oncological interventions have proven their efficacy in reducing emotional distress, the fear of recurrence, and post-traumatic symptoms, while facilitating better psychosocial adjustment to the diagnosis and contributing to an increase in health-related QoL [3–5]. A common factor among some of these interventions is the group component [6, 7]; throughout an oncological process, social support emerges as a crucial factor. Studies suggest that patients lacking a social support network undergo more challenging healthcare experiences [8]. Moreover, variables such as emotional distress and post-traumatic growth have been found to be related to social support [9–11]. On one hand, as highlighted by Gonzalez‐Saenz de Tejada et al., social support plays a role in reducing emotional distress [11]. On the other hand, prior research has found a positive association between higher levels of social support and increased post-traumatic growth [9, 10].

Within the broader framework of social support, one specific form is “peer support.” Peer support (PS), as defined by Hu et al., is a process in which individuals with the same illness come together to exchange information, share experiences, and provide mutual support and encouragement to face and overcome difficulties [12]. Although research on the impact of PS on oncology patients does not lead to conclusive results, BC patients are often interested in knowing the experience of other patients who undergo similar situations [13, 14]. Some studies suggest that PS can create a safe space leading to emotional support and mutual understanding [15]. Furthermore, interaction among patients beyond the support group can alleviate feelings of loneliness and isolation [16]. The evidence supports the notion that PS may play a significant role in the emotional well-being and QoL of women with BC. Participation in support groups and communities can provide significant benefits, such as improvement in searching, obtaining, and understanding information, a greater perception of social support, an increase in recognition through the sharing of experiences, and even the learning of coping strategies [17, 18]. The symbiotic relationship between improving information-related skills, perceiving increased social support, recognizing shared experiences, and learning coping strategies ultimately leads to a sense of empowerment within the community [17–19]. Additionally, empowerment-based interventions in cancer patients have been shown to promote post-traumatic growth [20] while also leading to a decrease in emotional distress through the positive influence of empowerment over autonomy in self-care, perceived control, and feelings of self-efficacy [21–23]. It also improves adherence to treatment, an issue of particular significance given that some treatments may be self-administered at home [21, 22, 24]. Furthermore, empowerment has been observed to encourage healthier lifestyles, enhance attendance rates for medical tests, and contribute to improved treatment outcomes [24–26]. Empowerment is in fact understood as a multidimensional concept of the awareness of one’s own strengths and the exertion of control over one’s [27].

As Zimmerman (1995) pointed out, it is useful to distinguish between empowering processes and empowerment outcomes, the latter being a consequence of the former. Empowerment processes refer to the actions and experiences that enable individuals to develop skills, manage resources, work as a team, expand social support networks, and strengthen leadership, all with the objective of gaining control and influencing decisions that affect their lives. On the other hand, empowerment outcomes are a product of these processes [28]. As previously found in the study conducted by van Uden-Kraan et al., patients diagnosed with BC and chronic illnesses who participate in online support groups may undergo processes such the exchange of experiences with other participants that contribute to empowerment outcome [19].

Traditionally, communities and support groups have been developed in face-to-face settings, which may not involve a professional facilitator [29] However, for over a decade, the use and development of the internet and social media have given prominence to online support groups and virtual patient communities, not only in the field of oncology but also in mental health and chronic illnesses [19, 29, 30]. Research regarding differences between face-to-face and online PS experiences has identified common factors as well as benefits associated with specific delivery formats [29]. Among the specific benefits, online communities offer the possibility of remaining anonymous, reducing implementation costs, and facilitating accessibility [27, 31]. Finally, it is essential to consider the digital literacy of patients to ensure that their participation is not hindered by difficulties in handling technology [32].

Regarding the potential benefits of PS, in 2019, the Catalan Institute of Oncology established a PS App for BC survivors called *Comunitats* in Catalonia and Oncommunities in Madrid (from now on in this article, *Comunitats*). The app aims to provide an accessible, secure, and anonymous space where BC survivors can receive PS. The community operates asynchronously, allowing conversations to extend over time, in contrast to the limitations of face-to-face support groups. Unlike other PS groups, *Comunitats* involves healthcare professionals such as psychologists, nurses, and social workers, and offers health education resources on topics that patients most frequently seek information about [13]. Given the potential impact of PS reported in previous research, this study aims to investigate whether (1) participation in *Comunitats* promotes empowerment among BC survivors; (2) empowerment processes within the community defined by Uden-Kraan [19] can predict empowerment outcomes; and (3) empowerment outcomes are related with other variables such as post-traumatic growth and emotional distress.

## Methods

### Design and participants

We conducted a prospective cross-sectional study, observing participants during their first 3 months in *Comunitats*. A total of 273 patients were initially referred to the community. After applying listwise deletion to handle missing data, we obtained a final sample of 121 breast cancer survivors, which ensured no missing values and preserved the integrity of the dataset. This sample includes women undergoing treatment and those in post-treatment phases, following different oncological treatment pathways. These participants were engaged in this virtual support community in Catalonia (*n* = 92) and Madrid (*n* = 29) between July 2019 and March 2023. Data were analyzed by using correlation and regression analyses, following the methodology outlined by van Uden-Kraan et al. [19]. The inclusion criteria for participants in the support community were the following: (1) a diagnosis of BC in any stage; (2) possession of a smartphone; and (3) signing the informed consent document. Participants were referred to the community by healthcare professionals—mainly nurses and psychologists—during consultations. Additionally, cancer patient associations were notified about the program so they could inform patients involved in their programs. Once patients agreed to participate, they were referred to the community coordinators. These professionals then contacted each person by phone to clarify any doubts and obtain informed consent. Moreover, they also guided the participant on how to download and use the app, as well as to complete the registration process on the platform. Additionally, information sessions were held in the hospital. For those who attended them, a designated time was offered to address any questions, as well as to sign the consent, and help to manage with the app if they were interested.

### Comunitats: virtual patient community

**Fig. 1:**
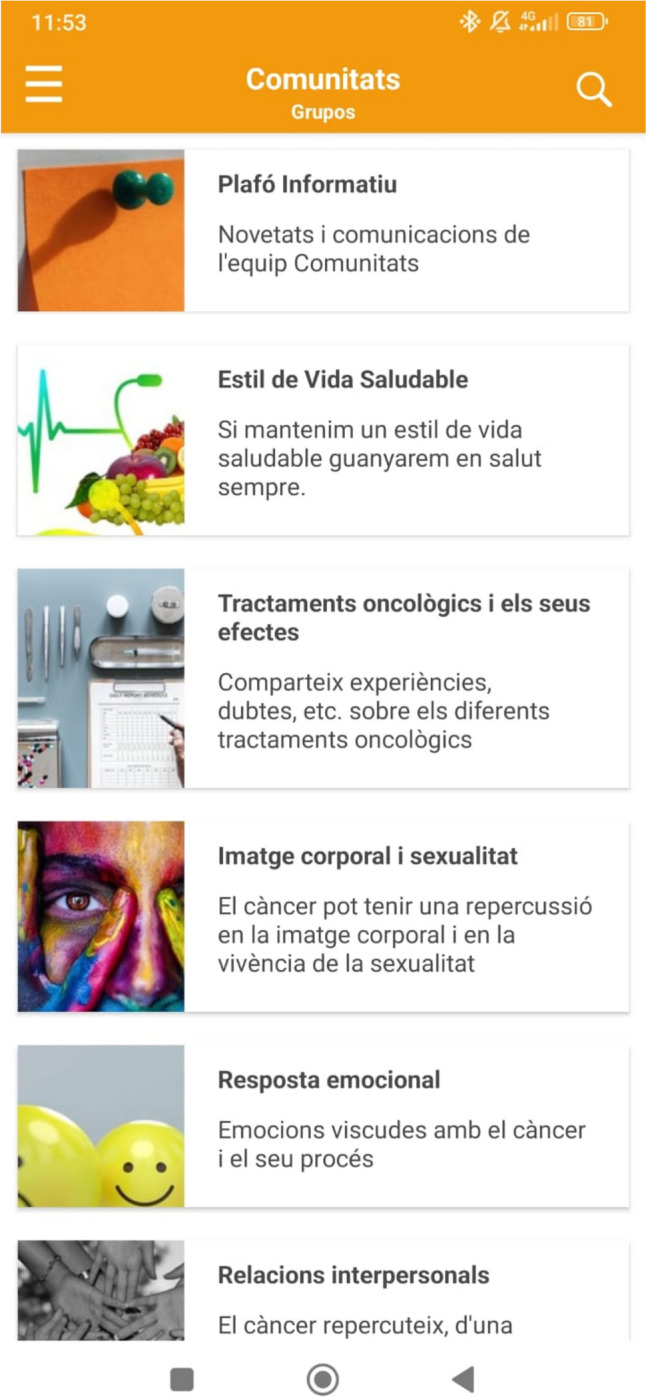
App’s interface

*Comunitats* is a virtual patient community associated with a governmental health department aimed at promoting PS, education, and emotional/social support among women diagnosed with BC. Through the community, users can interact anonymously and asynchronously in various chat rooms that are categorized into nine subjects (see Table 1). In addition, to facilitate PS, the chat rooms are monitored and dynamized by healthcare professionals, including psychologists, nurses, and social workers, to promote participation and to address technical questions asked by users. Also, users in each chat room have access to psychoeducational resources related to the chat’s subject of interest, such as information videos, adapted scientific information, or infographics. Figure 1 shows the app’s interface.

**Table 1:** Chat rooms included in *Comunitats*

Chat rooms topics	Content information
Information board	Information about activities linked to cancer and healthcare
Healthy lifestyle	Health promotion and well-being, such as exercise or diet
Oncological treatments and its effects	Clinical cancer information, such as different oncological treatments and their adverse effects
Body image and sexuality	Body image and sexuality during and after oncological process
Emotional response	Emotional and coping issues related to BC
Interpersonal relationships	Social and family relationships
Life changes after cancer	Life changes after cancer, including positive or negative changes
Employment issues, social, and community resources	Work, sick leave, social activities, and workshops
Technical issues	Reporting of any technical issue with the App

### Variables and instruments

Online assessments were conducted via the secure online assessment tool Qualtrics (Qualtrics, Provo, UT) at two time points: at the time of inclusion in the community (T1), and 3 months after inclusion (T2). The following data were collected:

#### Sociodemographic and clinical variables

At baseline (T1), sociodemographic variables such as age, marital status, employment status, and education were assessed. Clinical variables related to psychological and oncological history were also recorded. At the second evaluation point (T2), updates on employment status and the clinical variables were requested.

#### Emotional distress

The Hospital Anxiety and Depression Scale (HADS) [33] evaluates distress in individuals facing physical illnesses. It comprises 14 items organized into two separate subscales for anxiety and depression symptoms, each scored on a 4-point scale from 0 to 3. Higher scores indicate increased distress, with scores under 10 interpreted as low distress, scores between 10 and 16 indicating moderate distress, and scores over 16 representing high distress levels. The reliability of the scale calculated with our sample was *α* = 0.89 for anxiety and *α* = 0.85 for depression. This scale was administered at T1 and at T2.

#### Post-traumatic growth

The Post-traumatic Growth Inventory (PTGI) [34] measures perceived positive changes in individuals after suffering a traumatic event. It consists of 21 items graded on a Likert scale from 0 to 5. This self-administered inventory allows individuals to rate the level of change experienced, associating higher scores with a greater perceived positive impact of the traumatic experience. Scores are categorized as follows: 0–14 indicating no growth, 15–46 suggesting possible growth, and 47–105 reflecting growth. The scale has been validated in Spanish with an oncological sample, and we also assessed its reliability within our sample, obtaining an *α* = 0.95. The PTGI was administered at T1 and T2.

#### Empowerment

Van Uden-Kraan’s Empowerment Questionnaire [19] was administered after the 3-month period of participation. It is a specific and comprehensive self-reported questionnaire that measures two main concepts:

- Empowering processes: referring to the events or actions occurring within online PS groups that lead to empowerment in their participants (e.g., exchanging information or sharing experiences). This part of the questionnaire is composed of 29 items, of which two were inapplicable in our study; the remaining 27 were administered. Each item is rated on a 4-point scale from 1 “rarely or never” to 4 “often.”
- Empowerment outcomes: consequences of participating in the community (e.g., being better informed or enhanced social well-being). This part of the questionnaire is composed of 38 items, scored on a 5-point scale from 1 “completely disagree” to 5 “completely agree.”

Additionally, an overall empowerment value was calculated by summing the individual scores of each empowerment outcome, resulting in a composite score that reflects the total empowerment experienced by the participants. The possible range of this composite score spans from a minimum of 7 (if all individual outcomes scored 1) to a maximum of 35 (if all individual outcomes scored 5).

Specific items related to both empowering processes and empowerment outcomes can be found in the supplementary material provided.

#### Participation in the online community

While actively participating in the online community, we collected use-related data from the app. The information gathered specifically includes the number of postings made by participants.

### Data analysis

The data were analyzed using the IBM SPSS Statistics 27.0 software (IBM corp., 2020). Shapiro–Wilk test was performed in the variables of interest and their distribution was inspected visually and found to be non-normal; therefore, non-parametric tests were used. After descriptive analyses of participants’ demographic and clinical data, correlations between empowerment outcomes, empowerment processes, emotional distress, and post-traumatic growth were assessed with Spearman’s rank correlation (Spearman Rho) test to guide subsequent regression analysis. To correct for multiple testing the Benjamini–Hochberg procedure was used. For the variables with a statistically significant correlation with empowerment processes and outcomes subscales (*p*-value < 0.05), partial correlations controlling for age and time since diagnosis were conducted. Finally, a multiple linear regression analysis was used to predict overall empowerment outcomes based on processes occurring in the online community, since emotional distress and post-traumatic growth variables did not yield significant values in partial correlations. Additionally, to assess the robustness of our findings, post hoc power analyses for the regression analysis using the G*Power software [35] were conducted.

## Results

### Sociodemographic and clinical characteristics

Among the 121 participating women, the mean age was 50.63 years (*SD* 7.27), and the mean time between diagnosis and joining the community was 3.39 years (*SD* 5.41). Baseline sociodemographic and clinical characteristics are listed in Table 2.

**Table 2:** Baseline sociodemographic and clinical characteristics of participants

	*N* = 121
Age mean (*SD*)	50.63 (7.27)
Years since diagnosis mean (*SD*)	3.39 (5.41)
Finished treatment *n* (%)	88 (72.73)
Marital status *n* (%)	
Married	82 (67.77)
Divorced/separated	20 (16.53)
Single	18 (14.88)
Widow	1 (0.82)
Education *n* (%)	
Primary education	5 (4.13)
Secondary education	9 (7.44)
Higher education	41 (33.88)
University education	66 (54.55)
Work status *n* (%)	
Active	25 (20.66)
Work leave	61 (50.41)
Unemployed	3 (2.48)
Passive	4 (3.31)
Retired	22 (18.18)
Other	6 (4.96)
Origin *n* (%)	
Catalonia	92 (76.03)
Madrid	29 (23.97)
Emotional distress (HADS) mean (*SD*)	16.44 (8.10)
Post-traumatic growth (PTGI) mean (*SD*)	50.15 (22.51)

Abbreviations: *HADS*, Hospital Anxiety and Depression Scale; *PTGI*, Post-traumatic Growth Inventory

### Participation in the online community

Regarding participation, 45.5% of participants (*n* = 55) published posts in the community, with an average of 11.93 (*SD* = 18.13) posts during the 3-month period. Significant differences (*p* < 0.05) were observed in age and years since diagnosis, with younger and more recently diagnosed individuals being more active participants.

### Empowerment processes and outcomes

Regarding the empowering processes, the most commonly reported processes were “exchanging information” and “finding recognition” (Table 3). Individual item scores indicated that the respondents perceived the information shared in the online groups as both understandable (81.8%) and usable (73.5%). However, 51.2% of participants found that the information provided in the community was new only sometimes, or even seldom. Two thirds (66.1%) of the survivors reported the feeling of “not being the only one.” To a lesser extent, the respondents also “shared experiences,” “helped others,” and “encountered emotional support” in the community.

**Table 3:** Mean scores for each empowerment process

	Mean (*SD*) *N* = 121
Exchanging information (1–4)	3.0 (0.81)
Finding recognition (1–4)	2.66 (0.91)
Sharing experiences (1–4)	1.99 (1.07)
Helping others (1–4)	1.91 (0.92)
Encountering emotional support (1–4)	1.39 (0.51)

As for the empowerment outcomes, the ones experienced most strongly were “being better informed” followed by “improved acceptance of the illness” (Table 4). Frequencies of separate items reveal that 62.8% of participants felt better informed as patients and had the feeling that they now had the knowledge needed to manage their illness. Additionally, 50.4% of participants reported an increased ability to seek help, while 48.8% felt more able to tell others when they were no longer able to do something. To a lesser degree, participants also reported feeling more confident about the treatment, increased optimism and control, enhanced self-esteem, feeling more confident in the relationship with their physician, and enhanced social well-being.

**Table 4:** Mean scores for empowerment outcomes

	Mean (*SD*) *N* = 121
Being better informed (1–5)	3.67 (0.88)
Improved acceptance of the illness (1–5)	3.35 (0.83)
Feeling more confident about the treatment (1–5)	3.27 (0.78)
Increased optimism and control (1–5)	3.24 (0.58)
Enhanced self-esteem (1–5)	3.16 (0.82)
Feeling more confident in the relationship with their physician (1–5)	3.07 (0.64)
Enhanced social well-being (1–5)	2.95 (0.83)
Overall empowerment (7–35)	23.37 (4.87)

### Relationships between empowerment and other related variables

All empowering processes during the interaction in *Comunitats* showed a significant correlation with both individual empowerment outcomes and overall empowerment (Table 5).

**Table 5:** Spearman Rho coefficients for the relationships between the processes that took place within the online community and the outcomes experienced by the participants

*N* = 121	Outcomes							
	Being better informed	Confidence with the physician	Confidence about the treatment	Improved acceptance	Optimism and control	Enhanced self-esteem	Enhanced social well-being	Overall empowerment
Processes								
Exchanging information	0.574**	0.437**	0.448**	0.480**	0.398**	0.372**	0.390**	0.595**
Encountering emotional support	0.281**	0.243*	0.354**	0.260**	0.263**	0.263**	0.301**	0.354**
Finding recognition	0.624**	0.399**	0.459**	0.486**	0.495**	0.441**	0.421**	0.594**
Helping others	0.314**	0.195*	0.251**	0.240*	0.253**	0.263**	0.269**	0.336**
Sharing experiences	0.407**	0.282**	0.362**	0.339**	0.328**	0.295**	0.454**	0.456**

*p < 0.05, ** p < 0.01

Concerning the relationship between empowering outcomes and post-traumatic growth and emotional distress, PTGI displayed significant weak to moderate correlations with “feeling more confident about the treatment” (*p* < 0.05), “increased optimism and control” (*p* < 0.01), “enhanced self-esteem” (*p* < 0.01) and overall empowerment (*p* < 0.05). HADS showed a negatively significant weak to moderate correlation with “enhanced self-esteem” (*p* < 0.05) and “increased optimism and control” (*p* < 0.05). However, in partial correlation analyses, neither PTGI nor HADS yielded significant results; as a result, they were excluded from the linear regression in order to streamline the model and enhance its interpretability by focusing on the most influential predictors of overall empowerment within the context of *Comunitats*.

In the regression analysis (Table 6), the findings suggest that the overall empowerment can be partially predicted by the processes taking place within *Comunitats*, as indicated by an adjusted R^2^ of 0.371. This implies that 37.1% of the variance in overall empowerment can be accounted for by the model. Notably, the most influential predictors of overall empowerment are “exchanging information” and “finding recognition.” The post hoc power analysis yielded a value of 0.999, indicating high statistical power of our results.

**Table 6:** Regression analysis results

Total empowerment outcome (*N* = 121)							
	Unstandardized coefficient		Standardized coefficient	*p*-value	*t*	*R*^2^	Adj. *R*^2^
	*B*	SE	*β*				
						0.397	0.371
(Constant)	12.633	1.551		< 0.001	8.147		
Exchanging information*	1.641	0.579	0.272	0.005	2.835		
Encountering emotional support	0.118	0.852	0.012	0.890	0.138		
Finding recognition**	2.010	0.556	0.372	< 0.001	3.615		
Helping others	− 0.346	0.550	− 0.065	0.531	− 0.628		
Sharing experiences	0.489	0.582	0.107	0.402	0.841		

*p < 0.01, **p < 0.001

## Discussion and conclusion

### Discussion

This study sought to examine the connections between empowering processes within *Comunitats* and the empowerment outcomes reported by participants. Additionally, the study aimed to identify variables that might predict more substantial empowerment outcomes.

In agreement with previous research [17–19], results of this study reveal that participating in the PS community increases the feeling of recognition through sharing experiences as well as an improvement in information and its management.

The most prevalent empowering process observed in interactions within *Comunitats* was “exchanging information,” as van Uden-Kraan et al. [19] reported in their study. As highlighted by Hu et al. [12], BC-related information provided by participants may be incomplete or biased due to a lack of training among peer supporters. However, as mentioned above, *Comunitats* offers psychoeducational resources and involves healthcare professionals who can correct information if needed or guide participants toward the appropriate information source if the question is beyond the scope of the support community. Survivors reported “finding recognition” in online community interactions, in agreement with prior research emphasizing that PS fosters mutual understanding and recognition through shared experiences [15, 18]. Based on our research findings, “helping others” is the process that occurred least frequently. Considering that 54.5% of the participants included never posted in the online community, this result may be attributed to the fact that lurkers (participants who read the chats but do not actively participate) cannot engage in helping others. However, the data collected on community participation do not allow us to ascertain whether survivors who did not post are lurkers or simply did not access the conversation groups. As a result, we cannot determine whether there is any difference between active participants and lurkers in this case.

In the line of findings of van Uden-Kraan et al. [19], the empowerment outcome most reported was “being better informed.” Prior research suggests that in PS groups, a predominant aspect of group participation is the exchange of information, encompassing the sharing of knowledge related to the disease, treatment methods, or recommendations [17]. Furthermore, although to a minor degree, “increased optimism and control” is also reported, mainly, participants reported an increased ability to seek help and felt more able to tell others when they were no longer able to do something. Surprisingly, and in contrast to van Uden-Kraan et al. [19], the least reported empowerment outcome was “enhanced social well-being.” This result may be influenced by the level of active engagement within the online community. Notably, fewer than 50% of participants posted during the 3-month period, and the average post count below 12. This contrasts with the study conducted by van Uden-Kraan et al. [19], which included a larger and more actively participating sample. A potential explanation for these differences in participation may be related to the age of the sample and the time since diagnosis, as inferred from the results, with the most active participants being younger and more recently diagnosed. Likewise, the sample studied by van Uden-Kraan et al. [19] included younger participants and more recent diagnoses compared to our own.

While no significant correlations were found between emotional distress, post-traumatic growth, and overall empowerment this may be attributed to the fact that participation in the support community does not constitute a structured intervention, unlike empowerment-based interventions that have shown stronger relationships in prior studies [20, 23]. However, both emotional distress and post-traumatic growth were significantly correlated with “increased optimism and control” and “enhanced self-esteem.” These findings are consistent with research suggesting that optimism and self-esteem are resources able to contribute to relieving emotional distress and increasing post-traumatic growth [36, 37]. Regarding the empowering processes undertaken in *Comunitats*, our results indicate a moderate capacity to explain the overall empowerment reported by participants. In particular, “finding recognition” and “exchanging information” emerged as the most influential predictors, in agreement with findings reported by van Uden-Kraan et al. [19]. Similar results were obtained in online support groups beyond BC; [38] found that information support and recognition positively influenced users’ confirmation of expectations about the community; confirmation of expectations was linked with the willingness to continue using the online community [38]. Hence, future research might explore the accomplishment of users’ expectations as a mediator for gaining a deeper understanding of the outcomes observed.

This study has certain limitations that should be considered when interpreting the results. First, the 3-month period imposes a time constraint that limits the assessment of the long-term impact of participating in the community. In addition, the sample size was limited to 121 survivors, and the inclusion only of women diagnosed with BC may restrict the generalizability of the findings to individuals with other types of cancer. Therefore, it is important to take into account the potential for bias in the sample, as well as the absence of a control group. Moreover, data about other activities, such as group psychotherapy or involvement in patients’ associations that may encompass components of PS are not available, a circumstance that might introduce a confounding variable into the relationship between participation in the community and the results observed. Furthermore, the participation measure was limited to a binary approach (i.e., whether participants did or did not make any posts) and the count of the number of messages, which prevented us from noting differences between lurkers (those who consume information without actively contributing) and active participants.

### Conclusion

The results indicate that participation in *Comunitats* promotes empowerment among BC survivors. The findings reveal that specific processes within the community, especially “exchanging information” and “finding recognition,” contribute to overall empowerment. However, these processes explain the empowerment outcomes only to a modest extent. Future research is required to explore additional factors influencing the outcomes reported by participants and to identify the characteristics of participants who might derive greater benefits from participating in the community.

### Implications for practice

Offering online peer support communities in oncological processes could help participants to feel more empowered. Empowerment positively impacts self-care autonomy, self-efficacy, and treatment adherence, promoting healthier lifestyles and enhanced treatment outcomes. Therefore, we recommend that health professionals encourage cancer patients to participate in peer support communities. Additionally, knowing that there are different community processes that foster empowerment, support professionals of the community should promote the exchange of information and mutual recognition among participants.

## Supplementary Information

Below is the link to the electronic supplementary material.

Supplementary file1 (DOCX 27 KB)
