# Supplementary material for: Empowerment among breast cancer survivors using an online peer support community
Source: Support Care Cancer. 2024 Dec 28;33(1):56. doi: 10.1007/s00520-024-09119-5 (PMC11680672; doi:10.1007/s00520-024-09119-5)
Supplement: Supplementary file 1 — Supplementary file1 (DOCX 27 KB) [file 520_2024_9119_MOESM1_ESM.docx]

**van Uden-Kraan’s Empowerment Questionnaire**

1. **EMPOWERMENT PROCESSES**

Answer categories: ‘seldom to never’; ‘sometimes’; ‘regularly’; ‘often’.

**Exchanging information**

The information and tips exchanged in this online support group are...

1. ... understandable
2. ... valuable
3. ... usable
4. ... new
5. ... applicable to my present situation
6. ... reliable
7. ... correct
8. ... of added value to the information I receive from my care providers
9. ... in line with the information I receive from my care providers

**Encountering emotional support**

Does it ever happen in this online support group.. .

1. ... that someone in this online support group is empathic?
2. ... that someone in this online support group consoles you?
3. ... that someone in this online support group pays you a compliment?
4. ... that someone in this online support group is interested in you?
5. ... that someone in this online support group pays particular attention to you in special cases, such as during illness or moving house?
6. ... that someone in this online support group reassures you?
7. ... that someone in this online support group offers you sound advice?
8. ... that someone in this online support group points out your strengths?
9. ... that someone in this online support group conﬁdes in you?
10. ... that someone in this online support group asks you for your help or advice?

Excluded items: ... that someone in this online support group invites you to have (personal) contact outside this online support group? ... that someone in this online support group starts a private conversation with you?

**Finding recognition**

Does it ever happen in this online support group that...

1. ... you recognize yourself in the stories of other online support group members?
2. ... you experience the sense of ‘not being the only one’?
3. ... others are an example to you?
4. ... you realize that you are not so bad off after all?

**Helping others**

Does it ever happen in this online support group that...

1. ... you can be an example to other participants?
2. ... you can offer advice and support to others?

**Sharing experiences**

Does it ever happen in this online support group that you can share...

1. ... your experiences with your illness with others?
2. ... your everyday experiences with others?
3. **EMPOWERMENT OUTCOMES**

Answer categories: ‘completely disagree’; ‘disagree’; ‘neither agree nor disagree’; ‘agree’; ‘completely agree’.

**Being better informed**

Through my participation in online support groups...

1. ... I feel better informed as a patient.
2. ... I understand my illness better.
3. ... I have a clearer picture about my illness.
4. ... I feel like I have more (correct) knowledge at my disposal to deal better with my illness.

**Feeling more conﬁdent in the relationship with their physician**

Through my participation in online support groups...

1. ... I feel better prepared for a doctor’s appointment.
2. ... I am more knowledgeable about which questions to ask my physician.
3. ... I can explain my needs to my physician better.
4. ... I have more courage to raise matters with my physician.
5. ... I am more able to oppose my physician.
6. ... I understand the information provided by my physician better.
7. ... the relationship with my physician has improved.
8. ... the relationship with my physician has deteriorated.
9. ... I am more able to judge when I really need the help of my physician.
10. ... I feel less dependent on my physician.
11. ... I am more able to think along with my physician about my treatment.

**Improved acceptance of the illness**

Through my participation in online support groups...

1. ... I am able to be more open about my own illness.
2. ... I can tell others more easily when I am no longer able to do something.
3. ... I can ask others for help more quickly.
4. ... I can give in to my illness better.
5. ... I can accept my illness better.

**Feeling more conﬁdent about the treatment**

Through my participation in online support groups...

1. ... I can stick to my treatment regime better.
2. ... I am more able to follow the medical guidelines and advice of my physician.
3. ... I know where to go with questions about my illness.
4. ... I feel I am more skilled at dealing well with my illness.
5. ... I feel able to make the right decisions with regard to my illness.

**Increased optimism and control over the future** (a = .76):

Through my participation in online support groups...

1. ... I feel more in charge of the course of my illness.
2. ... I feel I can inﬂuence my illness myself.
3. ... I feel more in control over what is happening to me.
4. ... I feel less in control over what is happening to me.
5. ... I feel that what happens to me in the future is to a large degree dependent on myself.
6. ... I have learned to be more positive.
7. ... I have more faith in the future.
8. ... I have less faith in the future.

**Enhanced self-esteem**

Through my participation in online support groups...

1. ... I have a greater sense of worth.
2. ... I have a more positive attitude towards myself.
3. ... I am in general more content with myself.

**Enhanced social well-being**

Through my participation in online support groups...

1. ... I feel less lonely.
2. ... I have made new social contacts.
